# Metabolomics Signatures of Aging: Recent Advances

**DOI:** 10.14336/AD.2020.0909

**Published:** 2021-04-01

**Authors:** Sunil S Adav, Yulan Wang

**Affiliations:** Singapore Phenome Centre, Lee Kong Chian School of Medicine, Nanyang Technological University, Singapore.

**Keywords:** aging, metabolomics, metabolites, lipids, amino acids, mass spectrometry

## Abstract

Metabolomics is the latest state-of-the-art omics technology that provides a comprehensive quantitative profile of metabolites. The metabolites are the cellular end products of metabolic reactions that explain the ultimate response to genomic, transcriptomic, proteomic, or environmental changes. Aging is a natural inevitable process characterized by a time-dependent decline of various physiological and metabolic functions and are dominated collectively by genetics, proteomics, metabolomics, environmental factors, diet, and lifestyle. The precise mechanism of the aging process is unclear, but the metabolomics has the potential to add significant insight by providing a detailed metabolite profile and altered metabolomic functions with age. Although the application of metabolomics to aging research is still relatively new, extensive attempts have been made to understand the biology of aging through a quantitative metabolite profile. This review summarises recent developments and up-to-date information on metabolomics studies in aging research with a major emphasis on aging biomarkers in less invasive biofluids. The importance of an integrative approach that combines multi-omics data to understand the complex aging process is discussed. Despite various innovations in metabolomics and metabolite associated with redox homeostasis, central energy pathways, lipid metabolism, and amino acid, a major challenge remains to provide conclusive aging biomarkers.

The proportion of the aged population is growing rapidly and it has been projected that the proportion of the aged people (over 60 years) in the world will increase to 2 billion by 2050[1]. Most importantly, the health of the aging population is declining and followed by an increase in the number of patients with age-related diseases. For example, the life expectancy of Singaporeans reached 84.8 years in 2017, became the world’s longest-lived nation in the world [2]. However, the life lived in ill health has also increased from 9.1 years in 1990 to 10.5 years in 2017. The higher rates of chronic diseases and disabilities have been noted in the aging population. Further, these aging populations remain more time in ill health across the population. Aging is not a disease, but the illed-aging population remains a severe challenge to the health care system. Although significant efforts are being taken in educating the aging population to maintain a healthy lifestyle, further attempts are needed to understand healthy aging, discover aging biomarkers, and develop anti-aging therapeutics to extend lifespan. Advanced emerging techniques in genetics, evolution, and biology can be integrated and used to investigate a critical aspect of aging [3].

Aging is a universal, natural, and multifactorial process characterized by an inexorable alteration in vibrant biological, physiological, neurological processes, and their progressive functional decline. These alterations may be programmed and controlled through switching “on” and “off” of certain genes as proposed in the theory of longevity [4] or due to cellular and molecular damages as explained in aging theories like free radicals, wear and tear, and basal metabolism [5]. The multiple theories including programmed longevity, endocrine, immunological, wear and tear, cross-linking, basal metabolism, free radicals, and many more have been proposed to describe the process of aging [4, 5]. These theories can be grouped into two main categories such as programmed loss of functionality and cellular damage related changes. These proposed aging theories inter-connect with each other in a very complex manner. The consolidation of the proposed hypothesis of “damage theories” links low energy basal metabolism to lifespan extension due to low reactive oxygen species (ROS) production rate, low molecular damage, and low oxidative damage [6].

Proteins, carbohydrates, lipids, and nucleic acids are four fundamental molecules of the living organism. The rate of decline in physiological functions of these molecules depends on several factors like lifestyle, diet, physical activities, structural and functional alterations in organs and systems, environmental exposure, and it varies with the individual [7]. Further, the functional decline at the molecular, cellular, and tissue levels, as well as oxidative damages, have resulted in increased susceptibility to and frequency of disease and hence advancing age is the major risk factor for age-related disorders [8]. Although, aging and age-associated disorders are mechanistically related, only some elderly people have an age-related disorder. Besides, chronic diseases cause a functional decline in cellular processes [9]. Hence, an appropriate approach that can effectively differentiate biological healthy aging and aging due to age-related disorders or other chronic diseases need to be adopted. An understanding of the aging process and the underlying mechanism is critically important in improving the quality of life and to grow old healthily as well as disease-free. Therefore, to discover and develop new interventions for the healthy aging and extension of lifespan, we must first understand the mechanism of aging. Further, we need to distinguish normal aging and age-related pathologies, and determine factors affecting healthy aging. More efforts are required to identify aging biomarkers that can predict biological than chronological age since biological age takes the individual physical and mental health into account [10]. The ideal biomarkers need to have a significantly strong correlation with age irrespective of disease or disease progression.

An advancing and emerging technology adopted in the discovery of biomarker remains “omics” since the omics-based platform can accommodate multi-parameter measurement and has greater potential in assessing multifactorial aging process. The “omics” technologies including genomics, proteomics, transcriptomics, and metabolomics, are being embedded with a powerful set of tools and concepts that allow us to dissect and scrutinize the functional network of genes, proteins, and metabolites present in a cell or organism. Omics technologies have discovered and identified hundreds of epigenetic mutations, gene expression levels [11], transcriptomics [12] protein expression [13, 14], post-translational modifications [15], and metabolite concentrations [16] that are associated with aging. Metabolomics is the newest ‘omics’ technology that focuses on obtaining an integral representation of the current metabolic status of an organism through a diverse set of metabolites associated with physiological and pathophysiological processes.

The metabolome is not directly involved in the central dogma of the information flow, unlike the genome, transcriptome, and proteome. A large number of metabolites, both endogenous and exogenous, that are the end product of genes and protein regulations can be measured using the metabolomic tools which further can determine the distinct physiological and/or pathological states and may depict the complexity of the aging process. Therefore, the main objective of this review was to provide recent advances in the metabolomic signatures of aging. Given the complexity of the metabolome and its dynamic nature, we highlighted age-associated metabolic changes and biomarker discoveries. We attempted to identify the correlation between metabolite concentration and aging. The main challenges and limitations in the field are discussed.

## State-of-the-art metabolomics technologies

Metabolomics is a rapidly emerging advanced high-throughput “omics” technology that complements data from genomics, transcriptomics, and proteomics in advancing our understanding of the biological system, and is highly applied in various fields including medicine, synthetic biology, plant metabolomics, and microbial system [17]. The genome, transcriptome, and proteome provides useful information, but cannot provide altered metabolome, a complete set of final functional metabolites [18]. On the contrary, metabolomics provides quantitative information of the identified metabolites within an organism, cells, or tissues, and it is the final “omics” level in a biological system. The metabolites are the final product of metabolism hence the altered metabolome highlights the effect of different factors including biological aging, pathophysiology as well as environmental factors [19]. The advances in technology and significant breakthrough in metabolomics research within the past decade offer valuable insights into the correlation of metabolite level with aging and diseases. Although metabolomics technology has high potential to elucidate the aging process, yet handicapped due to a lack of comprehensive annotated endogenous metabolite database to support the identification of as many metabolites as possible. The availability of pure compounds for the quantification of the identified metabolites and their validation are limited. It is still difficult to assign and differentiate the changes in the identified metabolites, whether they are solely due to neutral age-associated changes or due to damages or associated diseases. Hence, an in-depth research is still required to establish the contribution of metabolomics in the aging process.

Recently, advanced technologies like high resolution nuclear magnetic resolution (NMR) spectroscopy, liquid chromatography-mass spectrometry (LC-MS), gas chromatography-mass spectrometry (GC-MS) have been adopted to profile biofluids including plasma, serum, and urine [20, 21]. Adopting NMR spectroscopy for biofluid metabolite analysis has the advantage of generating robust and reproducing results, but it is less sensitive than mass spectrometry. Adopting complementary NMR and MS-based metabolomics may reveal a more complete profile of the metabolites. However, a lack of a well-established comprehensive, electronically accessible global database is a major limitation in the identification and quantification of urine metabolites. According to recent human metabolome database 4.0 (HMDB 4.0), the total number of metabolites is 114,100, of which 18,609 metabolites can be detected and quantified (https://hmdb.ca/statistics#biospecimen-statistics) [22]. The total number of metabolites identified and quantified in blood, feces, and urine are 18,061, 1,008, and 2,041 respectively. As per the statistics of Urine Metabolome Database (UMDB-http://www.urinemetabolome.ca) total metabolites and total proteins in urine are 4345 and 4560. UMDB database is a comprehensive, web-accessible resource that contains a total of 2651 confirmed urine metabolites [20], whereas HMDB comprises about 2,041 [22].

### Metabolomics of aging and aging biomarkers

Metabolomics is thought to be a useful system approach to study and understand the aging process since it has the potential to present an unbiased metabolite profile resulting from a coordinated physiological response to various intrinsic and extrinsic factors [23]. The critical role of metabolic pathways including the age-associated decline in mitochondrial and endocrine function, signaling pathways, and the role of caloric restrictions in the aging have been reviewed by Barzilai et al [24]. Recent studies [25, 26], have established the link between aging and nutrient sensing, lipid, and amino acid metabolism, and redox homeostasis. Metabolism can lead to the accumulation of by-products, which could cause cell damage. Therefore, type, heterogeneity, and properties of accumulated damages need to be investigated to establish a link between such damages and aging.

### Plasma metabolites biomarkers of aging

Blood is a connective tissue of the circulatory system, transporting absorbed nutrients to cells and collecting and carrying waste products from all parts of the body to the appropriate excretory organs. Blood plasma contains thousands of metabolites collected from all parts of the body [22]. The cellular and physiological processes in cells of different tissues and organs uniquely adjust in response to various factors, which can be reflected in blood plasma metabolome through metabolite levels. Analogous to a specific disease, aging cells, tissues, and organs may present an altered plasma/serum metabolite composition and hence these biofluids can serve as a golden source of the aging biomarker. At present, about 18,690 confirmed metabolites have been established in the human blood metabolome [22]. Adopting a non-targeted profiling approach to profile plasma\serum metabolome at different stages of age, different diet, and lifestyle, etc may provide the profile of thousands of valuable small molecules, that have the potential to be an aging biomarker. Moreover, an ideal biomarker irrespective if it's an aging or disease biomarker, should not only be sensitive and predictive, but it should be measurable in a readily accessible and less intrusive biofluid. The plasma/serum can be easily obtained with less invasive techniques. Therefore, establishing quantitative detection of plasma/serum metabolites may help to establish the link between the metabolic alterations and the aging process. Restated, human blood plasma\serum is a rich source of information that reflects individual differences in health, age, diet, lifestyle, and disease, etc., through metabolites level and composition.

Nicotinamide adenine dinucleotide (NAD+) is a redox coenzyme central to energy metabolism, found in every cell and involved in hundreds of metabolic processes. Accepting and donating electrons interconverts NAD+ with its reduced form NADH which plays a critical role in biological processes like cell death, cell signaling, DNA repair, gene expression, aging, [27], as well as in central carbon metabolic pathways such as glycolysis, the Krebs cycle, oxidative phosphorylation, fatty acid oxidation [28]. The compromised NAD+ level can have grave consequences on these processes and defects in energy production. Given the significance of the NAD+ in a variety of biological processes and pathways, the plasma and multiple tissues including the liver, adipose tissue, heart, brain, kidney, pancreas, lungs, spleen, muscle, and skin concentration was examined and noted a decrease in NAD+ level with age [27-29]. The plasma NAD+ level which remain at nanomolar concentrations, significantly lower than tissues, has declined sharply with age in the plasma samples of healthy humans across a wide age range from 20 to 87 years [30]. This study also noted a reduced NAD+/NADH ratio in the plasma of aged individuals. Escalating evidence confirmed the age-dependent decrease of NAD+ in plasma as well as in multiple organs, in the aging model (*Caenorhabditis elegans)*, different species of rodents, and humans [27, 30-34]. In conclusion, the age-dependent decrease in NAD+ has been noted in biofluids and different organs which supports NAD+ as a hallmark of aging. However, the depleted NAD+ level has also been detected in major neurodegenerative diseases, such as Alzheimer’s disease (AD) and Parkinson’s disease (PD), cardiovascular disease, and muscle atrophy [27]. Thus, an in-depth research is required to differentiate the age-dependent and age-associated disorder dependent decrease in NAD+ level.

In mammals, NAD+ is synthesized from a variety of dietary sources as well as from its major precursors such as tryptophan (Trp), nicotinic acid (NA), nicotinamide riboside (NR), nicotinamide mononucleotide (NMN), and nicotinamide (NAM). The long-term administration of NMN demonstrated an increase in energy metabolism, insulin sensitivity, lipid metabolism, mitochondrial oxidative metabolism, and protection from age-associated functional decline [31]. NR is a natural NAD+ precursor and beneficial in safeguarding against aging and age-related diseases [35], restoring mitochondrial dysfunction [36], promoting longevity, and also can be converted into NAD+ [36, 37]. In age-associated disorders like AD and PD, NR improved memory, learning, and motor functions [36, 38]. In rodent models of AD, the administration of NMN significantly decreased AD-associated β-amyloid (Aβ) pathology, restored mitochondrial functions, and protected neuronal cell death [39-41]. Aman et al. [38] have reviewed the key roles of NAD+, NAD+-synthesizing, and -consuming enzymes in aging and age-related diseases, and NAD+ as a therapeutics for extending lifespan. In brief, the NAD+ level could be a potentially good biomarker of aging and its biology could be fascinating for developing therapeutics for extending life span and treating several diseases. The approaches including supplementation of NAD+ or NAD+ precursors, activation of NAD biosynthetic enzymes, and inhibition of NAD+ degradation could be very useful to keep NAD+ at baseline. The supplement of not only NAD+ but also its precursors Trp, NA, NR, NMN, and NAM could be beneficial to extend lifespan.

Reactive oxygen and nitrogen species (RONS) are produced by several endogenous and exogenous processes. RONS are highly reactive molecules that cause oxidative deterioration of DNA, protein, and lipid. The free radical theory which is also termed as “oxidative stress theory of aging” is based on the structural damage to DNA, protein, and lipid [42]. The basal levels of RONS are crucial for redox signaling, cell survival, tissue homeostasis, immunity, stressor responses, and inflammation; but a high level is injurious to cells, causes damage to macromolecules, and contributes to aging and pathogenesis of age-associated disorders [43]. However, the oxidative stress is the consequence of the failure to maintain the physiological redox steady state, which is the self-correcting physiological response to different stresses. The NAD\NADH and NADP\NADPH redox couples are essential for maintaining cellular redox homeostasis [44]. The imbalance between RONS production and antioxidant defenses leads to the accumulation of RONS-induced damages [45]. Hence, maintaining the equilibrium redox homeostasis is considered as a golden means of life and healthy aging [46]. Recently Grant et al [34] infused 750 mg NAD+ over a 6 h period and noted the reduction in plasma activities of enzymes of hepatic stress. Thus, keeping the plasma NAD+ level at a good physiological level reduces stress and possible damage.

Lipid acts as a metabolic messenger, coordinates fat synthesis, storage, and catabolism, and creates a complex metabolic network to balance nutrient availability and energy demands [47]. The human plasma lipidome is composed of thousands of different lipids dissected into several major classes based on their structures [48]. The low-density lipoprotein (LDL) particles are the main carriers of sphingomyelins and ceramides, while the role of triglyceride and sphingomyelin species has been identified in insulin resistance ([49] and in AD [50], and many other diseases. Lipids play a central role in energy metabolism, as structural elements in cellular membranes and in signaling cascades both directly (signaling molecules- diacylglycerol, fatty acids, phosphatidylinositols, sterols, ceramides, and sphingolipids), and indirectly via prenylation or palmitoylation [51]. Being a key element in several biological processes, altered lipid metabolism may influence these processes significantly. The fat oxidation declines with age, that causes body fat to accumulate and its immediate consequence is to increase plasma free fatty acid concentration and or the non-oxidative disposal of free fatty acids. The age-associated increase in levels of phosphatidylcholinediacyl, phosphatidylcholine acyl-alkyl, lysophosphatidylcholine acyl, acylcarnitines, amino acid, etc was noted in a population-based study that included the KORA (Cooperative Health Research in the Region of Augsburg, South Germany) F4 study from Germany as a discovery cohort (with 1038 female and 1124 male participants in the age range of 32-81years) and the Twins UK study as replication (with 724 female participants) [16]. Particularly, seven metabolite concentrations (C0, C10:1, C12:1, C18:1, SM C16:1, SM C18:1, and PC aa C28:1) increased with age while histidine decreased, which can be interpreted as an incomplete mitochondrial fatty acid oxidation and altered cell membrane composition as a function of age. The metabolite concentration of unsaturated fatty acids, saturated fatty acids, nucleotides, carnosine, ergothioneine, and amino acids in the rat plasma at different ages was correlated with aging [52]. The concentrations of all saturated fatty acids e.g *i.e*., palmitic acid, stearic acid, myristic acid, and nonacosanoic acid, overall, increased during aging. While the concentrations of unsaturated fatty acids i.e., linoleic acid, oleic acid, palmitoleic acid, arachidonic acid, linolenic acid, docosahexaenoic acid, eicosapentaenoic acid, and eicosatrienonic acid, was decreased in elderly rats compared with young [52]. The decrease in unsaturated fatty acids might be the result of free radical accumulation.

Non-targeted metabolomic analysis of plasma samples from a cohort of 269 individuals were performed by using a GC-MS and LC-MS [53] and it was observed that more than 100 metabolites related to amino acid metabolism, citric acid cycle intermediates, oxidative stress markers, and nucleic acid metabolism were significantly altered with age. Further, these authors observed lower levels of carnitine, cholesterol, fatty acids such as linoleic acid and arachidonic acid that are related to lipid metabolism. Auro et al [54] profiled serum metabolites in 26,065 Finnish and Estonian participants of Northern European ancestry and found that the age-specific metabolic fingerprints differ significantly by gender, but with significant differences in the lipoproteins, cholesterol, and TG levels with age in both genders. Phosphoglycerides, sphingomyelins and phosphatidylcholine, and other cholines, all related to lipoprotein assembly showed association with age and gender. Similarly, in another study by Montoliu et al [55], sphingomyelins and phosphatidylcholine were projected as a putative marker as well as modulators of healthy aging. The plasma metabolite profile of 150 healthy humans ranging from 30 to 100 years of age were profiled by using high-throughput LC-MS technologies and specific metabolites such as pyruvate, α-keto-acids, α-hydroxy acids, lactate, niacin, choline, lysine, glucose, and phosphatidycholines, etc were projected as biomarkers that define the condition of long-living [56]. In particular, lipid species, predominantly a hydroxyl fatty acid (25-hydroxy-hexacosanoic), a polyunsaturated fatty acid (eicosapentaenoic acid), two phospholipids (phosphocholine [42:9] and phosphoserine [42:3]), and a prostaglandin (15-keto-prostaglandin F2α) decreased with age, indicating lipid and their metabolism are closely linked to the aging process. Similarly, using different long-lived mice models, metabolites associated with phosphatidylcholine metabolism were projected as biomarkers of an extended life span [57]. The plasma metabolome of a larger cohort of 2,327 was profiled by LC-MS and it was found that several polar compounds and lipid analytes were associated with longevity [58]. This study found the close link between citric acid cycle intermediate, isocitrate, and the bile acid, taurocholate, with longevity. However, the altered level of the citric acid cycle with longevity was established through modulating cardiovascular risk, whereas taurocholate was independent of either cardiovascular or cancer risk. Restated, some metabolic pathways associated with longevity are distinct from those involved in the development of diseases.

Lipid metabolism and especially lipoprotein size was suggested to play an important role in longevity. In a Leiden longevity study that included Caucasian families consisting of long-lived siblings together with their offspring and the spouses of the offspring, Vaarhorst et al [59] investigated the association of lipid levels and lipoprotein particle sizes with human longevity and found that offspring had larger LDL particle sizes and lower triglyceride levels. Further gender-specific analysis indicated an association of the LDL particle size with the male, whereas triglyceride levels are associated with female longevity. Similar observations were noted in the study of longevity in Ashkenazi Jews [60]. The plasma lipidome analysis in 1526 middle-aged offspring of nonagenarians (59 years ± 6.6) and 675 (59 years ± 7.4) controls showed an encouraging lipid metabolism marked by larger LDL particle size in men and lower total triglyceride levels in women. These studies established the association of LDL particle size with males, whereas triglyceride levels were associated with female longevity, which indicates that the role of lipid metabolism in longevity differs in men and women.

There are a large body of research on the metabolic profiles of plasma\serum. However, blood metabolite that contains both non-cellular (plasma or serum) and cellular components are very much limited. Red blood cells (RBCs) undergo a natural aging process occurring in the blood circulation and their lifespan is about 120 days [61]. Chaleckis et al [62] adopted an untargeted quantitative analysis technique to analyze the blood of 15 young (29 ± 4 y of age) and 15 elderly individuals (81 ± 7 y of age) to profile metabolite associated with age. This study found remarkable age-related alterations in the levels of metabolites, including 1,5-anhydroglucitol, dimethyl-guanosine, acetyl-carnosine, carnosine, ophthalmic acid, UDP-acetyl-glucosamine, *N*-acetyl-arginine, *N*_6_-acetyl-lysine, pantothenate, citrulline, leucine, isoleucine, NAD^+^, and NADP. As RBCs serve a crucial function in blood and hence based on fifty-five RBC-enriched metabolites, this study emphasized the importance of RBS metabolome in human aging research.

Several foodstuffs are enriched with diverse profiles of metals through bioaccumulation from water to crops, fish, and farm animals. The diet can be considered as the main exposure route to metals, though there are several other potential routes of exposure to these elements. Many essential metal ions are present in small amounts, some are nutritionally essential, while others are nonessential. Metals like copper (Cu), zinc (Zn), iron (Fe), and manganese (Mn) play a vital role in cellular and physiologic processes including catalytic, regulatory, and signaling, but in higher concentrations or over-consumption, they might represent a health risk. Elements such as cadmium (Cd), arsenic (As), mercury (Hg), and lead (Pb) in daily foods remain toxic. After entering an ecosystem, some metals induce a wide range of physiological, biochemical, and behavioural dysfunctions through their contribution to oxidative stress and neurological disorders and play a critical role in the aging process as hypothesized by the metal ion theory of aging [63]. By adopting inductively coupled plasma mass spectrometry (HR-ICP-MS), Liu et al [64] determined the plasma concentration of 16 trace elements in children of age group 3-12 years and found higher concentration in children of age 3-6 years with some exception [Rubidium (Rb), Strontium (Sr), Selenium (Se), Cadmium (Cd), and Arsenic (As)] in boys. Age and gender impact the plasma levels of trace elements. Age-related changes in plasma were associated with behaviour, dietary habits, and lifestyle. In the Italian population, the blood Cu level was not influenced by age [65], however, other studies found either increased[66] or decreased [67]level of Cu with age. However, the concentration of blood Zn was positively correlated with age[65]. The limited literature exists on the impact of essential trace elements on the aging process.

### Urine metabolites biomarkers of aging

Urine is a chemically complex biological fluid that remains an attractive biospecimen source for biomarker study since it is non-invasive, easily obtainable, largely free from interfering proteins and lipids. Urine contains a high concentration of urea, inorganic salts, creatinine, ammonia, organic acids, and other water-soluble metabolites [20]. Although urine is a biological waste material, it contains the end-products of a wide variety of consumed foods, drinks, environmental contaminants, bacterial by-products, drugs, and a wide range of endogenous metabolites, and it can provide time-averaged information on the metabolic events that have occurred. Hence, the analysis of urine can provide a fingerprint of personalized endogenous metabolite markers that can be interpreted for age, gender, disease, diet, toxicity, etc. Diagnostically, the use of urine dates to ancient times where a brownish color of urine was considered as indicative of jaundice, a red hue color as urinary tract tumors, while the absence of color would be diabetes [68, 69]. Even in today's advanced technological era, the dipstick is used to test glucose, bilirubin, urobilinogen, hemoglobin, ketone bodies, nitrates, and proteins in the urine [70], and hence urine is an incredibly important biofluid to modern medical practice.

To discover age-related metabolic changes, Gu et al [71] employed NMR spectroscopy and analyzed the urine of children (12 years and below) and identified metabolites including creatinine, creatine, glycine, betaine, trimethylamine-N-oxide (TMAO), citrate, succinate, and acetone that correlated with age. Of these metabolites, the urine concentration of the creatinine increased with age while others were declined with age. Similarly, profiling urine metabolites of young and old (with 40 years as a cut-off) revealed significant changes in the metabolites like carnitine, 3-hydroxyisovalerate, creatinine, alanine, and trigonelline between young and old age groups [72]. In the investigation of the metabolite changes with age from 23 to 74 years, Psihogios et al [73] linked TMAO, citrate, phenylalanine, creatinine, and hippurate with age, while citrate, creatinine, trimethylamine N-oxide, glycine, creatine, and taurine were associated with gender. Urine metabolite analysis revealed an increase in the concentration of the hippuric acid, a kynurenic acid salt, ferulic acid sulfate, and suberic acid with advancing age, while acetate, fumarate, oxaloacetate, pyruvate, and trans-aconitate were decreased in the aged rats [74]. The urinary concentration of creatinine and hippurate were higher in younger subjects while TMAO and citrate were higher in older subjects. Hippurate is a glycine conjugate of benzoic acid and is related to the gut microbiome [75] and correlated to kidney function [76]. Glycine plays a major role as a neurotransmitter or biosynthetic intermediate [77], TMAO generated from choline, betaine, and carnitine via gut microbial metabolism and acts as an osmolyte in muscle tissues [78], creatinine has been linked to muscle function [79], betaine plays a vital role in the cardiovascular system [80], while citrate and succinate are TCA cycle intermediates, reflecting mitochondria function. The decrease in these Krebs cycle intermediates indicates alterations in mitochondrial activity. Restated, urinary concentration of these metabolites provides a metabolic fingerprint of the aging process and altered levels may impair several pathways.

In the cross-sectional Karlsruhe Metabolomics and Nutrition study, the urine metabolome of 301 healthy men and women aged 18-80 years were examined, and sedoheptulose, intermediate in the pentose phosphate cycle, was proposed as an aging biomarker in men [81]. These authors found a higher concentration of metabolites, 4-hydroxymandelic acid, glutaric acid, creatinine, N-acetylaspartic acid, and sedoheptulose in younger men, whereas 2,5-furandicarboxylic acid, hippuric acid, citric acid, 3-aminoisobutyric acid, and quinolinic acid had a higher concentration in older men. Collino et al [82] adopted a combined approach comprising NMR profiling and targeted LC-MS for phenotyping of longevity in female centenarians, elderly, and young individuals, and found increased excretion of phenylacetylglutamine (PAG) and p-cresol sulfate (PCS) in the urine of centenarians. The increased level of PAG and PCS suggests that the longevity process deeply affects the function and composition of the human gut microbiota. In short, urine analysis revealed that energy metabolism, lipid, amino acid metabolism, and gut microbiota functionality represent a key regulatory process in the human aging process.

The investigation of the age-associated metabolic profiles of urine in two distinct human populations i.e. Taiwanese (n= 857; age 54-91 years) and an American population (n= 1148 age 35-86 years) revealed some common and some distinctive features in these population [83]. This study observed a positive correlation of 4-cresyl sulfate (4CS) and PAG with age, whereas creatine and β-hydroxy-β-methyl butyrate (HMB) was negatively correlated with age in both populations. It’s important to note that the HMB is a metabolite of leucine and also serves as a precursor for cholesterol synthesis in muscle tissue [84]. Thus, it has both roles to protect and strengthen muscles. It also up-regulates protein synthesis in muscle tissue [85]. In short, in the pool of large urine metabolites that are impacted by age, creatine, and HMB metabolites are associated with muscle turnover, which declines with age, and hence it can be interpreted as potential biomarkers of aging which are reflective of a decrease in muscle mass with age.

Wang et al [86] monitored urinary metabolic profiles throughout the lifetimes of control-fed and diet-restricted dogs and found that urinary excretion of creatinine increased with age, reaching a maximum between ages 5 and 9 years and declining thereafter. The decline in creatinine has been linked with muscle turnover, which declines with age [87] and is considered as an index of muscle mass and is further directly linked with body mass [88]. The reduced physical activity, dietary changes, changes in gut motility, and immune competence can affect both the health and aging process. An exercise that includes physical activities improves health and reduces adverse effects of aging [89] as well as diminishes several psycho-somatic risks, including cardiovascular disease, diabetes, mild-to-moderate depression, etc [90]. Besides the level of creatinine, age-associated changes in other metabolites like arabinose, methylamine, myoinositol, pantothenic acid, threonine, gamma-aminobutyric acid (GABA), and leucine were noted in the urine of exercising Wister rats [91]. In addition to physical exercise, caloric restriction, altered exercise programming can be considered as an intervention to positively affect healthy aging. The supplementation of HMB can strengthen muscles, increase lean body mass, increase lifespan, and improve the lives of the elderly.

Urine is a necessity in several urological, metabolic, biochemical, nutritional, toxicological, general behavioral, and physiological studies in rodents. Rodents mostly rely on chemical communication for the directive of social and sexual interactions, and such chemical entities alter with age and could be a potential aging biomarker. In rodents, animal age can be characterized by urine scent which is contributed by volatile chemical entities. The particular urine scent assists female to distinguish male’s age [92]. To identify the association of the urinary metabolite concentration with age, Osada et al [92] analyzed the urine metabolite of 3-10 month and 17-month-old mice and established an age-based association of urine metabolites. This study demonstrated that the attraction of females to the odor of male mouse urine is greater when the urine is from aged males. The urine metabolite, 6-hydroxy-6-methyl-3-heptanone (6H6MH3O) is a volatile mouse sex pheromone and plays a major role in social and sexual communication [93, 94]. The mice with knockout of the flavin-containing monooxygenase 5 gene, Fmo5, slows metabolic aging via pleiotropic effects. Analyzing the urine of such knockdown C57BL/6J mice, Osada et al. [95] discovered a significant decline in the urinary concentrations of 6H6MH3O in males. Varshavi et al [96] performed metabolic profiles of urine from male, wild-type C57BL/6J and Fmo5-/- (FMO5 KO) mice and found the urinary concentration of 6H6MH3O changes with age, regardless of genetic background. The other identified metabolites were altered only in the FMO5 KO, or only in the wild-type mice, indicating the impact of genetic modifications on mouse aging. Elevated concentrations of urinary taurine were observed with age but only in wild type mice. The urine concentration of 6H6MH3O which is also associated with the citric acid cycle, fatty-acid, amino-acid, and nucleotide metabolism [96, 97]. In summary, mouse sex pheromone plays a major role in rodent communication that changes with age and can be a potential hallmark of aging. It is also associated with central energy metabolic pathways.

Aging biomarkers based on biological processes such as oxidative stress, protein glycation, DNA methylation, inflammation, cellular senescence, and hormonal deregulation were discussed and reviewed [98, 99]. Most of the aging theories consider oxidative stress as the driver of aging. Due to C-8 position's vulnerability to ROS and its mutagenic potential, 8-oxo-7,8-dihydro-2′-deoxyguanosine (8-oxodGsn) and 8-oxo-7, 8-dihydroguanosine (8-oxoGsn) remains the most studied DNA oxidative product which are promising biomarkers of aging [100]. A urinary 8-oxodGsn and 8-oxoGsn are molecules that may reflect the oxidative state of the whole body rather than a specific organ. The urine of 1,228 healthy Chinese residents (613 males and 615 females) 2-90 years of age were analyzed by LC-MS for the quantitative estimation of the concentrations of 8-oxodGsn and 8-oxoGsn, and an age-dependent increase in their level was observed [101]. The previous studies by these and other authors [102-104] also showed an age-dependent increase in these two biomarkers in mice, rats, and monkeys. In short, the urinary concentration of 8-oxodGsn and 8-oxoGsn can be a novel aging biomarker.

The oxidative stress has been linked to protein modification including Glycation, oxidation, nitration, and crosslinking of proteins and implicated in aging and age-associated disorders [105]. Accumulation of advanced glycation end products (AGEs) has been documented in aging persons where they play a critical role in the loss of bone density and muscle mass with age [106]. Deamidation of amino acid asparagine and glutamine has been proposed as a timer of biological events such as protein turnover, development, and aging [107]. The post-translationally modified glycated and oxidized proteins are directed for proteolysis which forms oxidized and glycated amino acid metabolites and excreted in urine [108]. To measure urinarily glycated, oxidized, crosslinked, and branched-chain amino acids, Masania et al [109] adopted the LC-MS/MS and profiled urine of 200 human subjects with early-stage health decline and healthy controls. These authors emphasized the potential use of these metabolites for a non-invasive health screening for early-stage health decline in metabolic, vascular, and renal health. The level of protein crosslink increased with age and contributed to the age-associated risk of Type 2 diabetes mellitus, cardiovascular disease, and chronic kidney disease [110, 111]. Glycated, oxidized and deamidated proteins result in protein aggregation [112] and have been implicated in aging and age-associated disorders [15, 113, 114]. These authors used brain tissue samples for their study. But urinary glycated, oxidized, crosslinked, and branched-chain amino acid quantification as performed by Masania et al [109] is lacking in aging research. The plasma levels of these modified amino acids are highly dependent on glomerular filtration rate and renal clearance [115]. Therefore, estimation of urinary levels of glycated, oxidized, crosslinked, and branched-chain amino acids could provide detailed information on aging processes, early-stage diagnosis of impaired metabolic, vascular, and renal disease.

By adopting an inductively coupled plasma mass spectrometry (ICP-MS), Bouatra et al [20] quantified a total of 40 metals in the urine samples. Similarly, Goulle et al [116] also adopted ICP-MS and validated identification and quantitation of 27 elements in plasma and 30 in the urine. However, these studies focused on the method development for trace element analysis, and their correlation with aging needs to be established. A wide range of metal ions including calcium (Ca), aluminium (Al), magnesium (Mg), and Se have been linked to age-associated disorders like AD [117]. Selenium and aging are closely linked and the role of selenium in aging and age-associated disorders has been reviewed by Cai et al[118], while the metabolomics of selenium is reviewed by Kazuo Suzuki [119]. The physical fitness of elderly patients is positively correlated with intake of phosphorus (P), Se, vitamin B6, C, D, E, niacin[120]. The basic and clinical studies have revealed an anti-aging effect of Se [118] owing to increase the antioxidant capacity of cells by enhancing the activity of superoxide dismutase and glutathione reductase. The trace elements, such as Zn, Se, and Fe, have a close relationship with longevity [121]. The dysregulated metal metabolism including Cu, Fe, and Zn have been linked with the neuropathology of AD, and enhance Aβ aggregation and toxicity, and accumulation of these metals in brain tissues of AD patients [122]. In short, Se supplementation has been proposed to have an anti-aging effect and its supplementation may extend life span and prevent aging-related diseases. However, well-designed studies on the impact of Se on the aging process are yet required.

### Fecal and Gut microbiota, and metabolite biomarkers of aging

Fecal matter primarily consists of microbial community biomass whose metabolomics characterization provides the metabolic relationship among the host, diet, and gut microbiota. Since fecal matter is dominated by microbial biomass, numerous researchers focused on characterizing microbial community structure using next-generation sequencing and metagenomics technique. However, with advancing technology, fecal metabolite analysis is also growing. The total number of fecal metabolites in the Human Fecal Metabolome DataBase (HFMDB) remains 6738 [123]. Of these total metabolites, 92% metabolites including short-chain fatty acids (SCFAs), medium-chain fatty acids (MCFAs), alcohols, amino acids, oligosaccharides, phenols, and polyphenol derivatives, sulfides, etc., were from gut microbial products [124]. Importantly, the development of gut microbiota begins at very early as soon as a new-born child is exposed to the environment [125]. After birth, the mode of feeding impact the gut microbiome, for example, the breastfed babies were found to have more *Bifidobacteria*, *Lactobacilli*, *Streptococci*, and *Staphylococci*, while the formula-fed child showed higher colonization of *Bacteroides*, *Clostridia*, and *Proteobacteria* [126, 127]. The reduced microbial diversity as well as intestinal commensal bacteria including *Bifidobacteria*, *Lactobacilli*, and *Bacteroides* have been linked to old age frailty and aging [128, 129].

**Figure 1.**
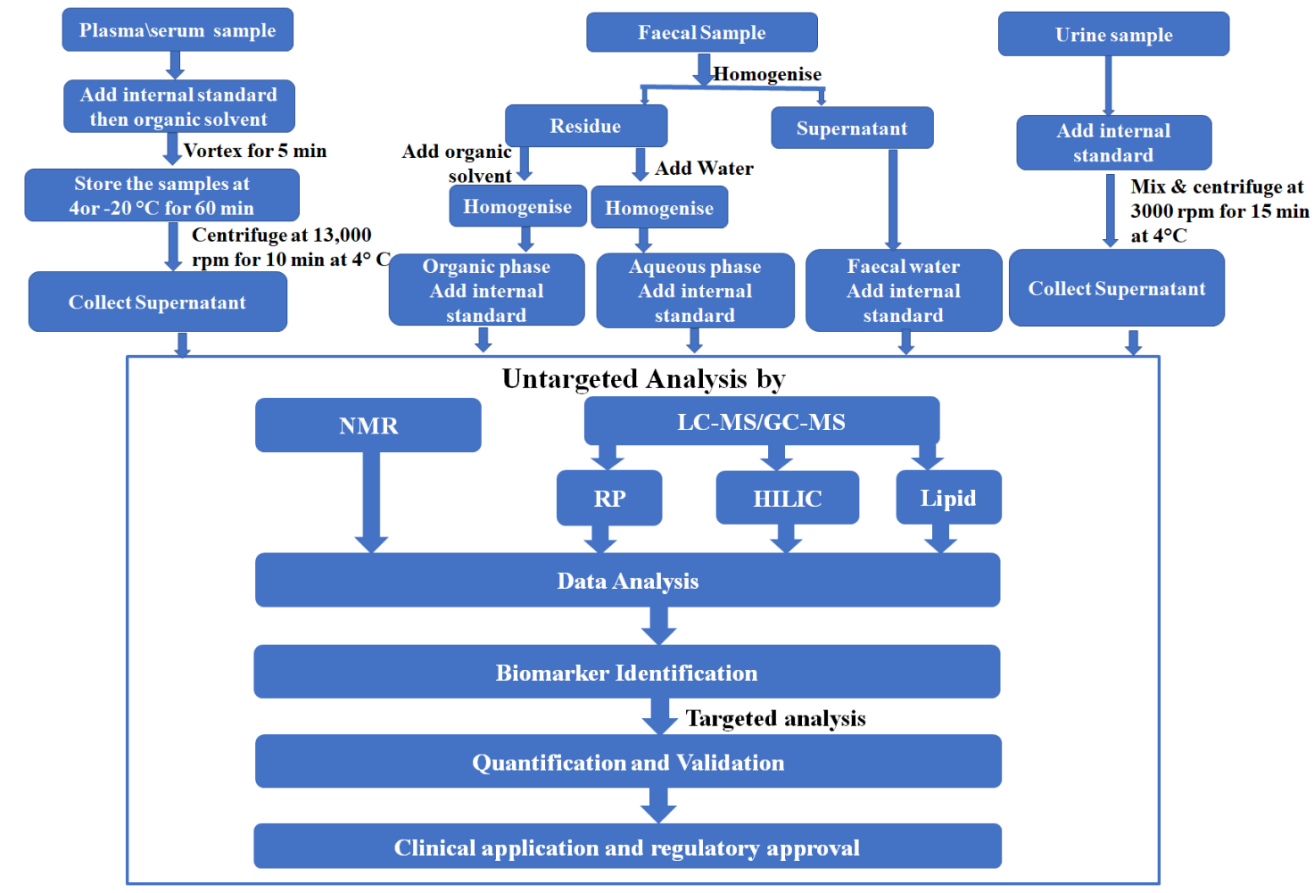
The simplified workflow of biofluids and fecal sample preparation for metabolomics analysis. LC-MS: Liquid chromatography-mass spectrometry; GC-MS: Gas chromatography-mass spectrometry; RP: Reverse Phase; Hydrophilic interaction liquid chromatography.

Gut microbiota contributes significantly to host physiological functions through hydrolysis and fermentation of non-digestible substrates, energy supply and harvest, production of vitamins, SCFAs, lipid metabolism and storage, amino acid synthesis, maintenance of intestinal barrier integrity, bile acid biotransformation systemic immune system, etc. [130-133]. Dysbacteriosis also called “Dysbiosis” in the gut microbiome and their metabolites have been linked to the abnormalities of gut barrier integrity and enhanced pro-inflammatory cytokines, which further trigger pathogenesis and progression of various metabolic diseases [134]. The disruption of balanced interaction between the gastrointestinal tract and the inhabiting microbes remains the cause of metabolic disorders, inflammatory bowel disease (IBD), allergy, obesity, cancer, diabetes, cardiovascular dyslipidemia, and neuropathology [135, 136]. As reviewed by Nicholson et al [134], gut microbiota regulates multiple host's metabolic pathways through metabolite production, giving rise to interactive host-microbiota metabolic, signaling, and immune-inflammatory axes that physiologically connect several organs including the gut, liver, muscle, and brain. Thus, the analysis of fecal metabolome has great potential in assessing host-microbiome interactions and it has become apparent that the gut microbiota and its metabolites are the crucial controller of host physiology, pathology, the aging process, and age-associated changes.

In humans, changes in gut microbiota during aging have been extensively studied [80, 134, 136-138]. The generalized outline of the fecal sample analysis is shown in figure 1. Lipid metabolisms remain critical in the aging process. To study the impact of age-related changes of microbiota on lipid homeostasis, Albouery et al [139] transferred the fecal microbiota of young vs. old donor mice into germ-free mice, allowed it to colonize for two months, and then analyzed, which showed altered lipids and fatty acids with the reduction in cholesterol. Considering the critical role of lipids in the neuronal membrane and cell signaling, the microbiota-driven alterations of lipids could greatly impact brain physiology. While in another study of aging mice model, Shenghua et al [140] observed a significant increase in the lipids including DG(14:0), DG(12:0), DG(8:0), TG(14:0), TG(16:1), PC(20:0), SM(d18:0), LysoPA(18:0), LysoPC(18:3), PA(18:4), MG(22:2). Calvani et al [141] found higher levels of 4-hydroxyphenylacetate and histidine, and lower concentrations of α-ketoisocaproate, α-ketoisovalerate, β-hydroxybutyrate, bile salts, isoleucine, and methionine in fecal samples from aged mice. Thus, the metabolic signature associated with aging were the changes in the levels of metabolites of crucial biochemical pathways, including amino acids and derivatives, short-chain fatty acids, intermediates of choline-betaine metabolism, nicotinate/nicotinamide metabolites, tricarboxylic acid (TCA) cycle intermediates, and ketone bodies. Due to the correlation between aging and the intestinal microbial community, the gut microbiota remains a potential target for developing novel strategies for healthier aging. The manipulation of the intestinal microbiome with host-friendly bacteria found in yogurt was recommended for healthy aging [142]. Similarly, Kau et al [143] emphasized the modulation of gut microbiota composition and diversity through diet and nutritional intake that can strengthen the innate and adaptive immune system, which could help in promoting healthier aging and also longevity. It is very challenging to derive the distinct fecal aging biomarkers since the lifestyles and diet significantly influence the assembly of gut microbial communities.

## Conclusions and perspective

Aging is a natural and very complex biological phenomenon dominated by an individual’s genomics, transcriptomics, proteomics, metabolomics as well as diet, lifestyle, and environmental factors. Metabolomics has the potential to link up genetic and proteomic variations to functional variations and provide innovative insights into metabolic, regulatory, and signaling activities in each cell or tissue. Recent technological developments and advances in high-throughput omics technologies have brought a revolution in technical innovations, insights in disease mechanisms, and significant developments in aging research. The individual omics data and the integration of data from a multi-omics platform including genomics, transcriptomics, proteomics, and metabolomics may provide novel insight into the aging process. In this review article, we reviewed metabolomic investigations related to the aging process and aging biomarkers in non-invasive biofluids like plasma, urine, and fecal matter or gut microbiome.

NAD+ plays a fundamental role in the metabolic reactions of a wide variety of metabolic pathways and it was found that its level in biofluids and various tissue declines with age. Hence, supplementation of NAD+ or its precursors was projected to be beneficial in extending lifespan. Keeping NAD+ concentration to youthful levels minimizes damages by the different stressors molecules and extend lifespan. However, further research investigations are required to improve our understanding of the potential crosstalk, and consequences on other pathways. Such research will add more values and strengthen support for targeted therapeutic interventions. Amino acids are fundamental structural molecules of life and an integrated component of functional and structural proteins. Amino acids are signaling molecules and can be used to alter the rate of aging. Amino acids such as leucine and arginine activate the target of rapamycin (TOR) and have the potential to alter lifespan. Glycine has been shown to extend longevity. The limited literature exists on the health benefits of other amino acids and needs more well designed and executed research in this area.

Lipids are of wide varieties and classes, and several hundreds of enzymes impact the lipid profile associated with the aging process. These enzymes have the potential to modulate the length, structure, and desaturation of FA and their incorporation into complex molecules that play a crucial role in signaling. Focusing these signaling pathways and altering them through genetic, dietary, or pharmacological interventions may provide a novel avenue of lipid metabolism and its modulation to extending lifespan. The caloric restriction substantially delays the aging processes. However, limited knowledge exists on the response as well as the impact of caloric restrictions on metabolic pathways involved in the maintenance and repair, nutrient sensing, cellular senescence, and longevity. The aging research mostly associated the aging process to cellular and macromolecular damage accumulation. Many types of stress and nutritional deficiencies further strengthen such damages and their accumulation can accelerate aging processes. However, limited literature exists on how these stresses including, oxidative, psychological, and mental health influenced the aging process.

In the past decade, substantial progress has been made in the detection of age-associated altered metabolites in non-invasive biofluids like plasma and urine. However, the list of a metabolite that alters with age is not conclusive and the main question in aging research remains unanswered. It is not clear how metabolite differences and which metabolite signatures can determine biological age and its modulation could extend lifespan. This could be due to complexity and heterogeneity among the individual and between the different organs. Another main limitation is the lack of a well-organized, global, unique metabolite database. To achieve a highly promising goal of understanding aging processes and aging biomarkers, the complex interactions of metabolites, altered pathways, regulatory networks need to be established.

The integration of data from the multi-omics platform including genomics, transcriptomics, proteomics, and metabolomics may provide a better understanding of the biological mechanism of aging. However, this integration of multi-omics data is challenging due to the complexity of annotation, mapping with pathways, network analysis, and interactions with environmental factors, such as diet and lifestyle. For the integration of multi-omics data from a different platform and sources, and international initiatives including the creation of an international network of technical centers that can process a large number of samples, equipped with advanced modern instruments, skilled technical experts, biostatisticians, expert geneticists, and biochemists are required. The single-cell genome-, transcriptome-, proteome- and metabolome-wide analysis remain less complex and integration of such data could eventually lead to the possibility of early determination of age, mechanism of the aging process, and aging biomarkers. The future of research lies in the clinical and biomedical applications to improve the life of an individual. To extend lifespan, anti-aging interventions into clinical practice may need a multidimensional systemic approach since aging is a multifactorial and complex process.
